# Contact model for DEM simulation of compaction and sintering of all-solid-state battery electrodes

**DOI:** 10.1016/j.mex.2022.101857

**Published:** 2022-09-13

**Authors:** Magnus So, Gen Inoue, Kayoung Park, Keita Nunoshita, Shota Ishikawa, Yoshifumi Tsuge

**Affiliations:** Department of Chemical Engineering, Faculty of Engineering, Kyushu University, 744 Motooka, Nishi-ku, Fukuoka 819-0395, Japan

**Keywords:** Fabrication, Plastic deformation, Mold compaction, All-solid-state battery

## Abstract

In this study, a discrete element method (DEM) that can simulate particle plastic deformation, sintering, and electrode compaction of all-solid-state batteries was developed. The model can simulate elastic, plastic, and viscoelastic deformations that occur particularly in mold compaction processes. When the stress exceeds the yield strength of the material, inelastic deformation occurs, which can be described by either a plastic or viscoelastic response. We applied this model to simulate mold compaction of an All-Solid-State Battery (ASSB) electrode. This study implements the following novel features:

• The model was derived from the Maxwell viscoelastic model and enabled the simulation of the elastic, plastic, and viscoelastic deformation of particles in a mold.
• Particle deformation and sintering are modelled by the rate expression of the equilibrium overlap.
• The area and spring factors are introduced to account for numerical issues when the porosity approaches zero.

## Graphical abstract

**Figure:**
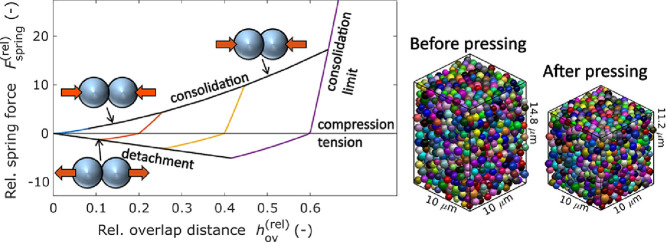

Specifications Table

**Table:** 

Subject Area:	
More specific subject area:	Battery fabrication simulation
Method name:	A discrete element model for deformation, sintering and mold compaction of battery electrodes
Name and reference of original method:	Discrete Element Method
	P.A. Cundall, O.D.L. Strack, A Discrete Numerical Model for Granular Assemblies, Géotechnique. 29 (1979) 47–65. https://doi.org/10.1680/geot.1979.29.1.47.
Resource availability:	N.A.

## Method details

The development of Lithium-ion batteries (LiBs) has paved the path for the development of electric vehicles which have tripled its sales over the course of recent two years 2019-2021 [11]. Despite its success, there is a market need for better safety, higher capacity and faster charging time. In all-solid-state batteries (ASSBs), the liquid flammable electrolyte has been replaced by inflammable solid electrolyte which increases the safety. Moreover, the capacity can be increased and the charging time can be significantly dropped. The core of an all-solid-state battery (ASSB) consists of an anode electrode and a cathode electrode which are separated by a solid electrolyte layer. Both the anode and the cathode consist of a mixture of SE and active material (AM) although the AM material usually differs.

### Introducing the equilibrium overlap

**Fig. 1:**
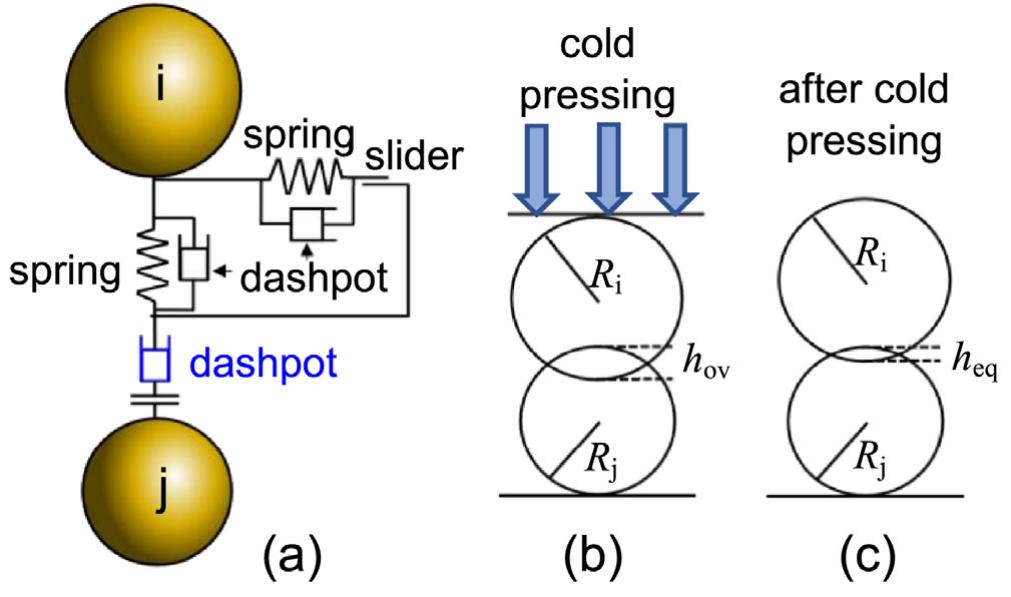
Schematics of the DEM force model that is reproduced and adapted from one of our earlier studies [1].

**Fig. 2:**
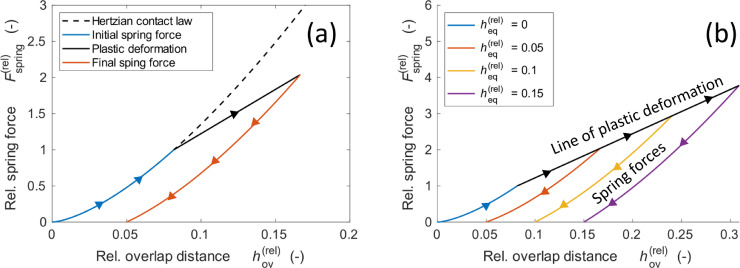
Force displacement curve during unloading and release for an individual curve (a) and varying maximum force (b).

We recently developed a new contact model of plastic and viscoelastic deformation [2] for ASSB electrode which is indicated by the blue dashpot in Fig. 1(a). The two particles under compression are shown in Fig. 1(b). As indicated in Fig. 1(c), the plastic deformation made the particles move toward each other even after release of the pressure. This deformation is expressed by the equilibrium overlap $h_{\text{eq}}$. Equilibrium overlap can also be observed in the loading and release curves in Fig. 2(a). The undeformed particles at the beginning follow the Hertzian contact law. However, when the spring exceeds a certain threshold, plastic deformation occurs. The relative spring force in the y-axis in Fig (a) is the ratio of the spring force to the threshold value. When the relative spring force exceeds unity, plastic deformation occurs, as indicated by the black line. After release, the curve of the spring force moved to the right with an equilibrium value above zero. The spring force is expressed as follows:

(1) $$F_{\text{spring}}=k_{n}\left(h_{\text{ov}}-h_{\text{eq}}\right)=4/3\cdot E_{\text{eff}}R_{\text{eff}}^{1/2}h_{\text{ov}}^{1/2}\left(h_{\text{ov}}-h_{\text{eq}}\right).$$

The effect of $h_{\text{eq}}$ on the force curves is shown in Fig. 2(b). The black arrow indicates the plastic deformation process, which we define as the dynamic change in the equilibrium overlap.

### Model derivation

Our plastic deformation model was derived from the Maxwell viscoelastic model where the stress was modelled based on the strain rates. Viscoelastic deformation means that, apart from an elastic response which stores energy, there is also a viscous flow response causing energy dissipation. For a constant stress, the Maxwell viscoelastic model [3] can be expressed as follows:

(2) $$\sigma =\eta \frac{\partial \varepsilon }{\partial t}=Et_{\text{rel}}\frac{\partial \varepsilon }{\partial t},$$

where η is the viscoelastic viscosity, ε is the strain, E is the Young's modulus, and $t_{\text{rel}}=\eta /E$ is the relaxation time. We developed a spring force equivalent version of this model by substituting $E=k_{n}/A$, $\varepsilon =\Delta h_{\text{eq}}/h$ and σ=F/A, resulting in the following plasticity force relation:

(3) $$F_{\text{plasticity}}=k_{n}t_{\text{rel}}\frac{\partial h_{\text{eq}}}{\partial t}.$$

In cold-pressing applications, plastic flow will not proceed unless a threshold force $F_{\text{th}}$ is reached. We assumed that the force from plasticity is equal to the excess force over the threshold: $F_{\text{plasticity}}=F_{\text{spring}}-F_{\text{th}}$; this results in the following rate expression for the equilibrium overlap during compression:

(4) $$\frac{\partial h_{\text{eq}}}{\partial t}=\frac{F_{\text{spring}}-F_{\text{th}}}{t_{\text{rel}}k_{n}}.$$

For viscoelastic simulations, $t_{\text{rel}}$ plays an important role in simulating creep in materials, as it is related to the viscosity of the viscoelastic material. For plastic deformation applications, $t_{\text{rel}}$ only has the role of a relaxation term, and we ensured that it is smaller than the simulation time, resulting in a steady-state simulation. The parameter $t_{\text{rel}}$ is important for viscoelastic simulations and the effect of this parameter on the force-displacement curve and on the compaction is investigated in Additional Information. In plastic deformation scenarios, the threshold force $F_{\text{th}}$ is nonzero and is calculated from the lowest yield strength $\sigma^{\left(\text{yield}\right)}$ of the contacting particles as follows:

(5) $$F_{\text{th}}=\text{min}\left(\sigma_{i}^{\left(\text{yield}\right)},\sigma_{j}^{\left(\text{yield}\right)}\right),\;\;A_{\text{con}}^{\text{eff}}$$

where $A_{\text{con}}^{\text{eff}}$ is the effective contact area. Because of the limiting information of the yield strength in the literature, we assumed the hardness, *H*, to be a reasonable approximation for $\sigma^{\left(\text{yield}\right)}$. In the initial stage of contact before plastic deformation, a parabolic stress profile can be assumed with a maximum stress that is a factor of 3/2 higher than the average, and the effective contact area can be calculated from the Hertzian contact as follows:

(6) $$A_{\text{con}}^{\text{eff}}=2/3\cdot A_{\text{con}}^{\text{spherical}}=2/3\pi \cdot h_{\text{ov}}R_{\text{eff}},$$

where $A_{\text{con}}^{\text{spherical}}$ is the spherical overlap contact area.

### The effect of sintering on contact force

Some materials with high deformability, such as LPS, can allow for room-temperature sintering at high pressures [4]. We modelled this sintering process by adding fusion contacts with cohesive interactions after pressing. If the overlap is less than the equilibrium overlap for a fusion bond, the spring force is negative, creating an attractive force. Herein, we also include plastic deformation in the tensile direction and there is a total of two plastic deformation processes: consolidation and detachment. Detachment occurs under high tensile stress under a negative spring force $F_{\text{spring}}<-F_{\text{th}}$, and the equilibrium overlap decreases under these conditions down to zero upon which the fusion bonds break. The rate of consolidation follows the expression

(7) $$\frac{\partial h_{\text{eq}}}{\partial t}=\left\{ \begin{array}{cc} \frac{F_{\text{spring}}-F_{\text{th}}}{t_{\text{rel}}k_{n}}, & \text{if}\;\;F_{\text{spring}}>F_{\text{th}}\\ 0, & \text{if}\;-F_{\text{th}}<\;F_{\text{spring}}<F_{\text{th}}\\ \frac{F_{\text{spring}}+F_{\text{th}}}{t_{\text{rel}}k_{n}}, & \text{if}\;\;F_{\text{spring}}<-F_{\text{th}}\;\left(\text{fusion}\;\text{bonds}\right) \end{array}.\right.$$

**Fig. 3:**
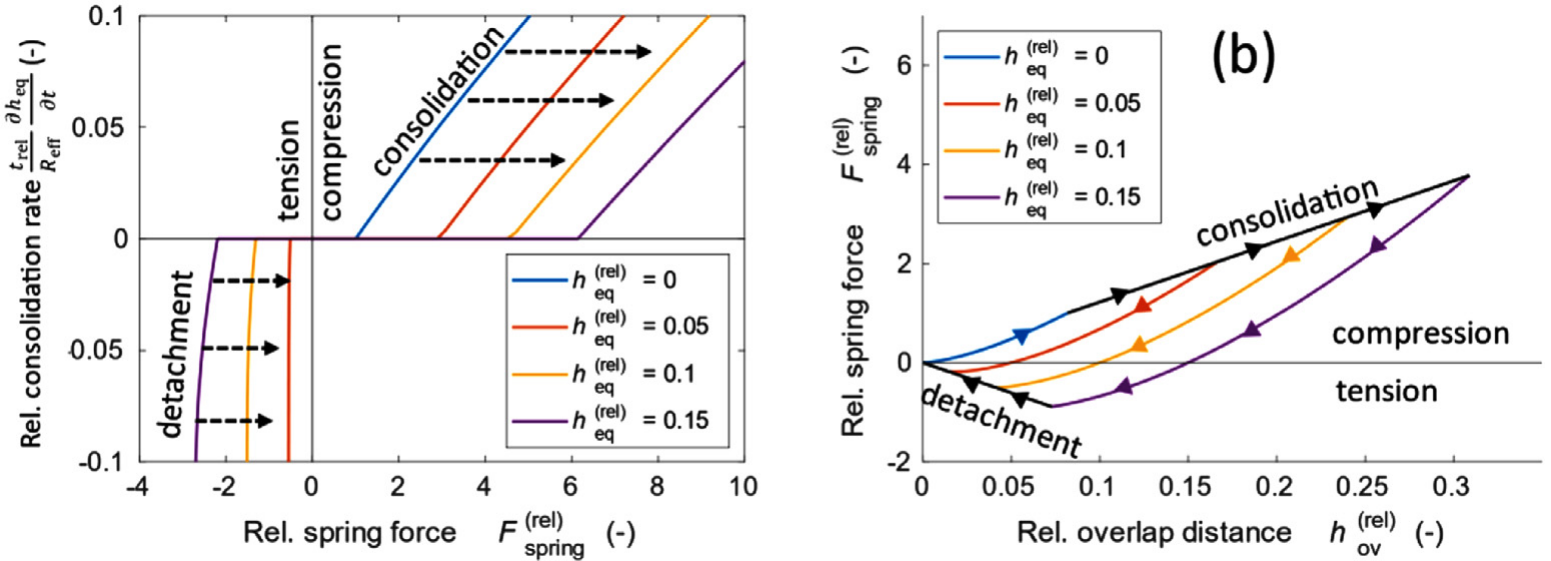
Plot of the relative consolidation rate with the spring force (a) and the interparticle force-displacement curve (b) including fusion bond contacts.

The relative consolidation rate is plotted with the relative spring force, as shown in Fig. 3(a). The consolidation rate increases during compression when the spring force exceeds the threshold, and detachment is caused by the tension. The force-displacement curve for the sintering process between the two particle pairs is shown in Fig. 3(b). Increased compression causes more consolidation, as indicated by the black arrow line. The degree of consolation can be quantified using $h_{\text{eq}}$. Particles that had been consolidated to a higher degree required a larger tension force for detachment into separate particles.

### The effect of the plasticity contact area on contact force

If plastic deformation proceeds, plastic creep causes the material to flow from the contact point, which further increases the contact area. Storakers developed a plastic contact model [5] which have been frequently applied to simulate mold compaction. However, his model cannot simulate elastic recovery because only plastic deformation is considered. We assumed that the area follows the Herzian contact law at small plastic deformations and inserted a factor term on the right-hand side in the following equation:

(8) $$A=A_{\text{Hertzian}}\left(1+c_{\text{area}}\frac{h_{\text{eq}}}{R_{\text{eff}}}\right)$$

**Fig. 4:**
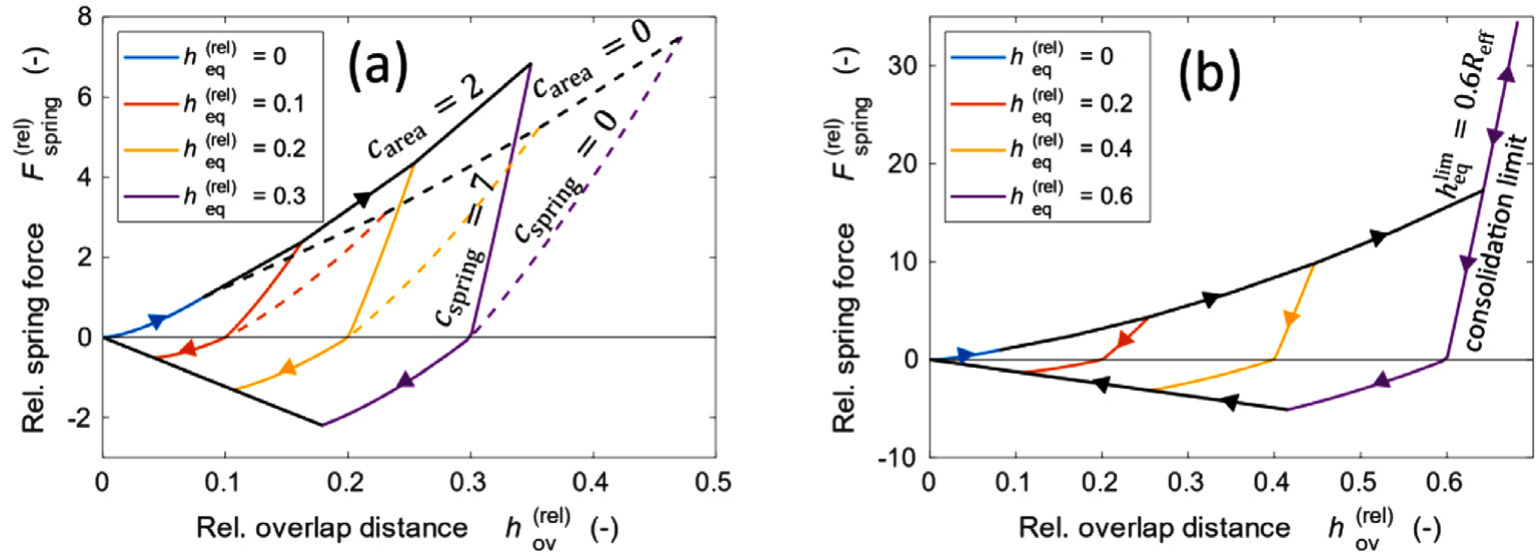
Force displacement curves that show the effect of $c_{\text{spring}}$ and $c_{\text{area}}$ (a) and with a consolidation limit $h_{\text{ov}}^{\text{lim}}$ (b).

The change caused by the Hertzian or plastic surface area is shown in Fig. 4(a). The area of contact between the particles becomes larger when the plastic creep from the contact point is considered.

When the contact area between each particle increased, the interparticle compaction stress decreases, and a higher mold pressure is required for continued compaction. We have previously used $c_{\text{area}}$ as a fitting parameter for fitting the packing density of the simulation to experimental results [1,6]. We also inserted a factor for the spring constant on the right-hand side in the following equation:

(9) $$k=k_{\text{Hertzian}}{\left(1+c_{\text{spring}}\frac{h_{\text{eq}}}{R_{\text{eff}}}\right)}^{\frac{3}{2}}.$$

We used 3/2 as the exponent because the spring constant increases both the area and with length contraction of the length. In addition, the growth of the spring force with the $h_{\text{ov}}$ follows an exponent of 3/2. The effect of $c_{\text{area}}$ and $c_{\text{spring}}$ on mold compaction is investigated further in section Method validation.

### Consolidation limit

In the literature, several methods have been proposed to overcome over-compaction. Some of these are referred to as Voronoi methods, where the local area is estimated from the volume of a polyhedron obtained from Voronoi tessellation [7]. There are several issues with these types of models. First, these methods are computationally expensive, as they require conducting Voronoi mapping followed by the volume calculation of each polygon. Our model employed a simple approach. Under high compression, the equilibrium overlap continues to increase until the maximum value $h_{\text{ov}}^{\text{max}}$ is reached. The parameter $h_{\text{ov}}^{\text{max}}$ determines the final packing density after cold pressing. As a default, we set $h_{\text{ov}}^{\text{lim}}=0.6R_{\text{eff}}$, which corresponds to the case where the solid volume fraction approaches unity when the simulation is run at a high mold pressure of 600 MPa. The force displacement curve with $h_{\text{ov}}^{\text{lim}}$ is shown in Fig. 4(b).

**Fig. 5:**
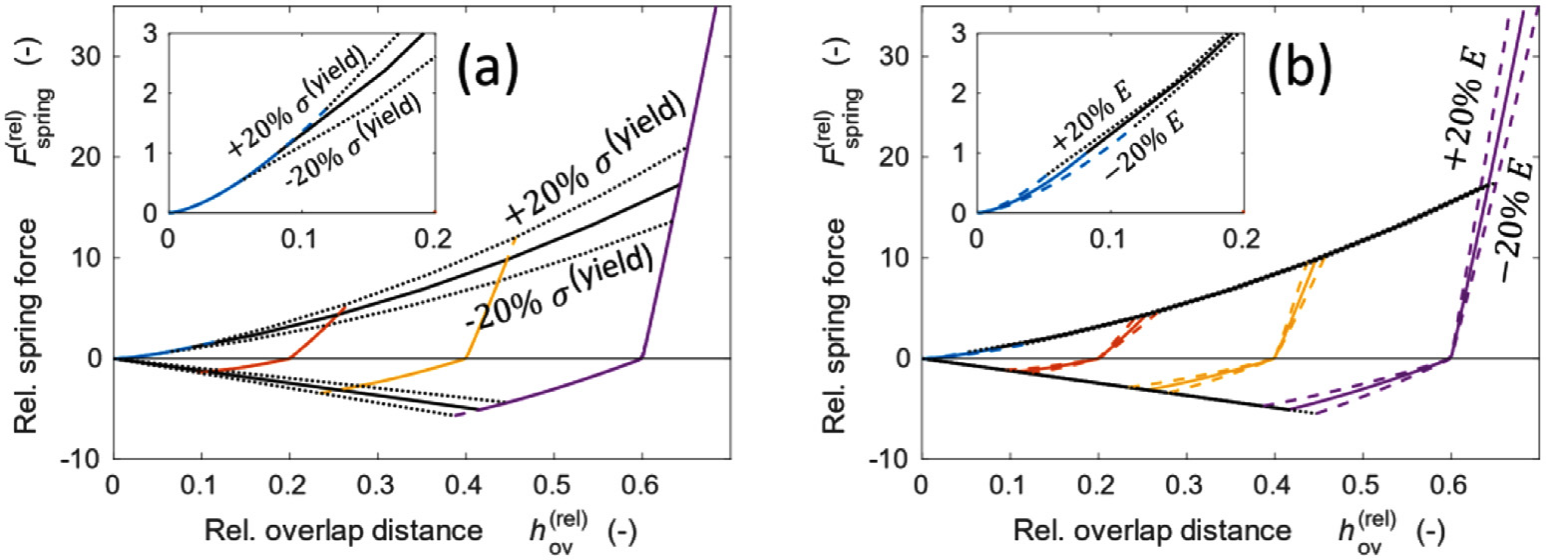
Effect of yield strength $\sigma^{\left(\text{yield}\right)}$ (a) and Young modulus E (b) on the force-displacement curve.

In Fig. 5, the effect of the yield strength $\sigma^{\left(\text{yield}\right)}$ (a) and the Young modulus E (b) on the force displacement curve for both small and large deformations is evident. While $\sigma^{\left(\text{yield}\right)}$ mostly affects the threshold force in the vertical direction of the force-displacement curve, E affects the curve in the lateral direction, and the effect of E on the threshold force is minimal.

### Method validation

**Fig. 6:**
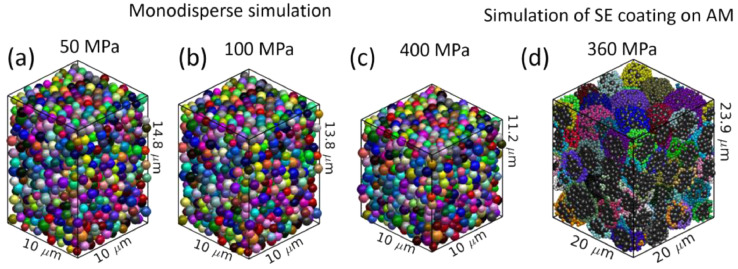
The particle distribution after simulation with different molt pressures (a-c) and application of our model to simulated SE coated AM particles as in our reference study [6].

We conducted a simulation of monodisperse LPS particles using the model described in the previous sections. The particle distribution after the pressing and release simulations is shown in Fig. 6(a-c). A monodisperse simulation was conducted to calibrate the parameters $c_{\text{spring}}$, $c_{\text{area}}$, and $h_{\text{eq}}^{\text{max}}$ in our simulations. In our reference study, we performed simulations of AM particles coated with SE, and the results after pressing are shown in Fig. 6(d). The key parameters used in this study are shown in Table 1. The restitution coefficient and the friction coefficient were arbitrarily assumed as a global value for all particle-particle pairs and particle-wall interactions. The hardness of the LPS material was previously determined by through instrumented indentation in mineral oil to prevent air exposure [8]. The parameters of the monodispersed and the coating simulation can be seen in Table 2. In the coated simulations, the AM and SE particles sizes and their relative volume fractions were obtained from Nakamura et al. [9].

**Table 1:** Material properties used in the simulations.

Symbol	Value	Units	Description	References
*e*	0.5	^−^	Restitution coefficient	This study
*E*_SE_	24	GPa	SE Young modulus (LPS)	Sakuda et al. [4]
*E*_AM_	199	GPa	SE Young modulus (LPS)	Cheng et al. [10]
*µ*	0.5	-	Friction coefficient	This study
*v*	0.3	-	Poisson ratio	This study
$H_{\text{SE}}$	1.9	GPa	Hardness of SE (LPS)	McGrogan et al. [8]
$H_{\text{AM}}$	11.2		Hardness of AM (NCM)	Cheng et al. [10]

**Table 2:** Simulation setup parameters in the different simulations.

Description	Monodisperse	Coated	Unit
Number of AM particles	0	10,746	—
Number of SE particles	1,846	27,263	—
Primary AM diameter	—	1	µm
Primary SE diameter	1	0.5	µm
Domain size	10	20	µm
Fraction of AM particles	0	0.734	—
AM aggregate size	—	5	µm
SE aggregate size	—	1.45	µm

**Fig. 7:**
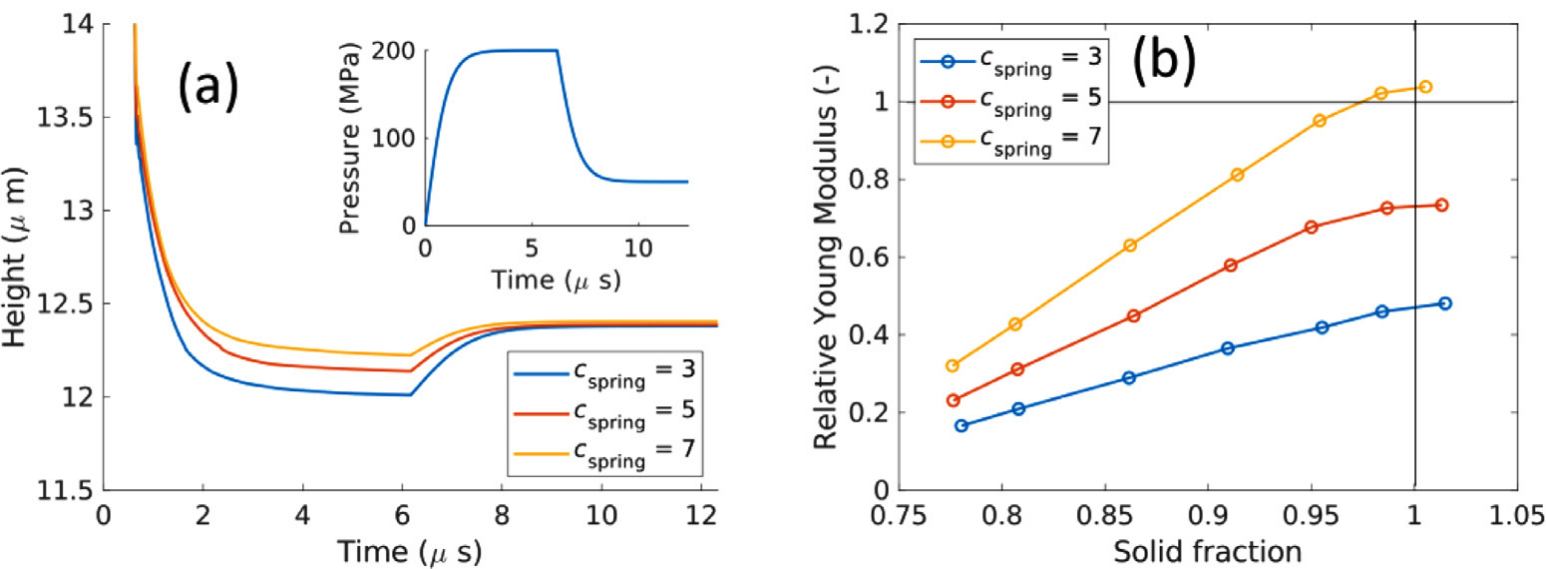
The effect of $c_{\text{spring}}$ on the dynamic change of the particle packing height (a) and the relative Young's Modulus.

The time series of the electrode height from the monodispersed simulation is plotted in Fig. 7(a) with different values of $c_{\text{spring}}$, and it is observed that the elastic recovery distance decreases with $c_{\text{spring}}$. The effective Young's modulus of the mold can be calculated from the change in pressure and the relative elastic recovery distance. We reasoned that when the solid fraction approaches unity, the effective Young modulus should approach that of the compressing material. In Fig. 7(b), the relative Young's modulus, $E_{\text{rel}}=E_{\text{electrode}}/E_{\text{LPS}}$ is plotted with the volume fraction. When the solid fraction approaches unity, the simulation with $c_{\text{spring}}=7$ resulted in an $E_{\text{electrode}}$ approaching that of $E_{\text{electrode}}$.

**Fig. 8:**
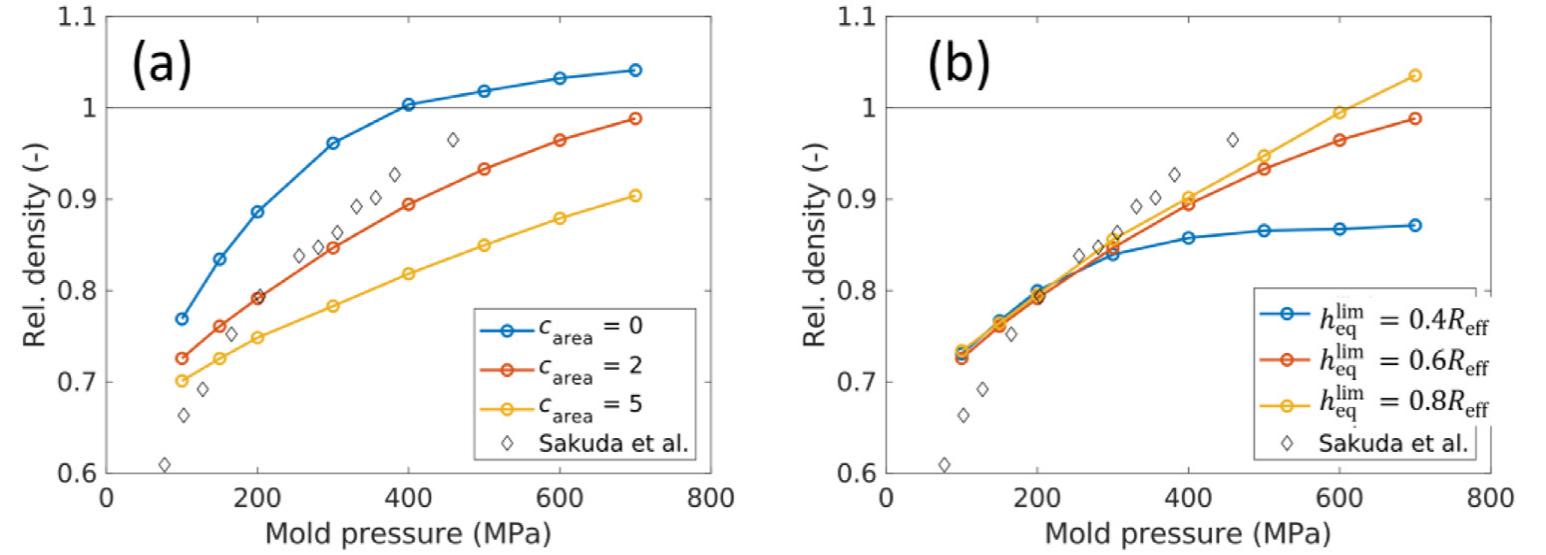
Plot of the relative density with the mold pressure and the effect of parameters $c_{\text{area}}$ and $h_{\text{eq}}^{\text{lim}}\;$on the relative density.

The effect of $c_{\text{area}}$ on the compaction is shown in Fig. 8(a). When $c_{\text{area}}$ increased, the electrode became more resistant to compaction. A good fit with the experiment of Sakuda et al. [4] was not possible at low mold pressures with this simple DEM model. This is because in this study, aggregates or agglomerates were not simulated as in some earlier studies [1,2,6], which could have increased the porosity significantly at low mold pressures. If the low mold pressure region is omitted, we can see that a value of $c_{\text{area}}=2$ gives reasonably good agreement with the experimental results. The effect of changing the consolidation limit $h_{\text{eq}}^{\text{max}}$ is shown in Fig. 8(b). As expected, higher values of $h_{\text{eq}}^{\text{max}}$ caused an increase in relative density. A value of $h_{\text{eq}}^{\text{max}}=0.6R_{\text{eff}}$ was chosen to be most appropriate because over-compaction at high mold pressures can be avoided for a reasonable fitting with experimental data.

Even though we compared and calibrated the compaction with experiments, more experimental studies may be needed in the future. Moreover, it was difficult to obtain material properties of the yield strength from the literature. Therefore, we used the hardness to calibrate our model. Although there is a dependence between the yield stress and the hardness, a different material might show a different relationship between these quantities which may errors in the prediction.

## Additional information

### Method overview and background

The discrete element method (DEM) is a Lagrangian solver developed by Cundall and Strack [12] that simulates the interaction between particles. This is ideal for granular materials as in ASSBs electrodes as it allows the prediction of stress localization between grains and deformation. In the reference paper to this study [6], DEM was utilized to simulate mold compaction of an ASSB porous electrode. Although our focus was on the battery manufacturing process, the simulation method can be applied to a wide range of applications such as pharmaceutical manufacturing.

To simulate the compaction process, a new model of plastic deformation was developed in a series of papers [1,^2^,6]. Although some explanations were given, we still concluded that a more in-depth explanation of the model is necessary to explain various mechanisms, its derivation, its applications, and to provide methods for other researchers to reproduce our results.

The original DEM model of Cundall and Strack [12], which is described section in the elastic contact mechanics section is based on a spherical overlap model and has been extensively used to simulate particle dynamics with elastic collision dynamics. However, in molds, plastic deformation cannot be ignored. There are several models that simulate plastic deformation while assuming negligible elastic deformation, such as the study by Storakers [5]. However, because we aimed to investigate elastic recovery, we aimed to develop a model that can simulate both elastic and plastic deformation. Several models have been developed to investigate dynamic systems where plastic deformation occurs from high energy collisions [13]. However, these models are not applicable in molds where the velocity is low and the dynamics is controlled by static interactions rather than the kinetic energy of the particles. In the literature, there are several elasto-plastic models that have introduced the concept of equilibrium overlap [7,14,15] in which the regular elastic dynamics are used, but the equilibrium distance between the particles is reduced owing to plastic deformation from compression.

The section Introducing the equilibrium overlap describes our the first version of our model for the ASSB electrode [2], where we adopted the equilibrium overlap concept to simulate the cold pressing process during fabrication. We developed a new method for calculating equilibrium overlap. During plastic deformation, plastic flow was assumed to follow the Maxwell viscoelastic model with a defined relaxation time that can be derived from the viscoelastic viscosity. A detailed explanation of the derivation can be found in the model derivation section.

In The effect of sintering on contact force section, we described how our sintering model functions and how fusion contacts develop during pressing and release. Only highly deformable materials, such as LPS, can form fusion bonds during cold pressing [4]. For our application of ASSBs [1,2,6] we allowed fusion bonds to develop during cold pressing for deformable LPS materials, but we did not allow this for AM materials such as NCM or SI. For AM, fusion bonding may exist initially within AM aggregates that have undergone sintering at high temperatures before cold pressing.

At high deformations in molds, the porosity approaches zero, and several phenomena contribute to the difficulty in adopting the spherical overlap approximation for the estimation of the contact area and spring constant. First, the areal contact between particles increases at a higher rate than that predicted by the spherical overlap approximation due to plastic deformation at the contact points. Second, as the particle shape changes, secondary contacts develop, as they are not detected by the spherical overlap model. Finally, the contact dependence is no longer valid, indicating that the force calculated from a given displacement is often underestimated [16]. In the reference paper of this study [6], our model has undergone several improvements to overcome these difficulties . In the implementation and the effect of plasticity contact area section, we introduced factors for the contact area and spring force that will enable a more accurate simulation of compaction and elastic recovery. In the implementation of the consolidation limit section, we set a limit on the interparticle equilibrium overlap to avoid over-compaction.

To describe our model in detail, we start with the fundamental model and gradually increase the complexity of the model and simultaneously explain the effect of the implementation on the force interaction. Finally in the application of model to particle DEM simulation section, we investigated the effect of the parameters on the compaction process in a DEM simulation.

### Elastic contact mechanics

When particles are in contact, they exert forces on each other. Fig. 1 illustrates the contact interaction between two particles *i* and *j* and includes a spring and dashpot in the normal and tangential directions. The blue dashpot denotes plastic deformation, which is explained in the following sections. The total particle-pair force between particles *i* and *j* is given by

(10) $$F_{i,\;j}^{\text{particle}-\text{pair}}=F_{i,\;j}^{\text{normal}}+F_{i,\;j}^{\text{tan}}.$$

The springs resemble the push-back force that the particle experiences upon contact. The dashpot resembles energy dissipation, which dampens movement. In the normal direction, the contact force is given by [17]:

(11) $$F_{i,\;j}^{\text{normal}}=\left(-F_{i,j}^{\text{spring}}+\eta \left(u_{i}-u_{j}\right)\cdot n_{i,j}\right)n_{i,j},$$

where $F_{i,\;j}^{\text{normal}}$ is the normal force, η is the damping coefficient, $u_{i}$ and $u_{j}$ are the velocities of particles *i* and *j,* respectively, and $n_{i,j}$ is the normal pointing from particle *i* to *j*. The damping coefficient can be obtained from the restitution coefficient *e* as follows [17]:

(12) $$\eta =-2\text{ln}\left(e\right)\sqrt{\frac{m_{\text{eff}}k_{n}}{\ln^{2}\left(e\right)+\pi }},$$

where $k_{n}$ [N m^−1^] is the normal spring coefficient, and $m_{eff}^{-1}$ is the effective mass given by

(13) $$m_{eff}^{-1}=m_{i}^{-1}+m_{j}^{-1}.$$

The spring force was obtained by

(14) $$F_{\text{spring}}=k_{n}h_{\text{ov}}.$$

where $h_{\text{ov}}$ is the overlap distance. The spring coefficient is a function of the particle radii and is based on a nonlinear Hertzian spring [18] given by

(15) $$k_{n}=4/3\cdot E_{\text{eff}}h_{\text{ov}}^{1/2}\;R_{\text{eff}}^{1/2},$$

where $R_{\text{eff}}$ [m] is the effective radius, and $E_{\text{eff}}$ [Pa] is the effective elastic modulus:

(16) $$R_{\text{eff}}^{-1}=R_{i}^{-1}+R_{j}^{-1},$$

(17) $$E_{\text{eff}}^{-1}=\left(1-\nu_{i}^{2}\right)E_{i}^{-1}+\left(1-\nu_{j}^{2}\right)E_{j}^{-1}$$

where $R_{1}$ and $R_{2}$ [m] are the neighboring particle radii, $E_{i}$ and $E_{j}$ [Pa] are the Young's moduli, and $\nu_{i}$ and $\nu_{j}$ [-] are the Poisson ratios. For the tangential forces, we used the Coulomb friction model. The slippage limit is imposed as follows:

(18) $$\left|F_{i,j}^{\text{tan}}\right|\leq \mu \left|F_{i,j}^{\text{normal}}\right|,$$

where the left-hand side is the static friction, the right-hand side is the kinetic friction, and μ is the friction coefficient.

### Application of the model to viscoelastic deformation

**Fig. 9:**
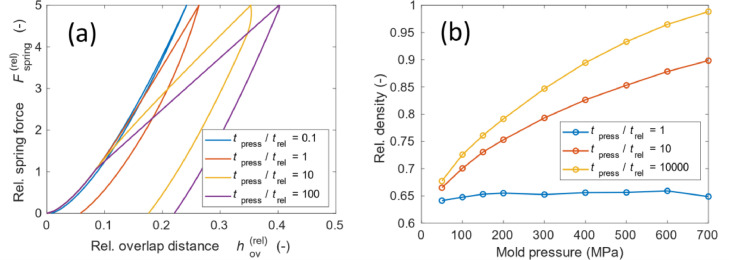
Effect of the pressing time versus the viscoelastic response time.

Although we focused on plastic deformation, the model can also be extended to viscoelastic deformation. In some applications, the viscoelastic relaxation time can be significant, where not only the magnitude of the forces is important but the time of the process in relation to the viscoelastic response time is important. Fig. 9 shows the effect of the pressing time versus the viscoelastic response time on the force curve (a) and compaction process (b). For batteries, this type of model can be applied to simulate Li, which has viscoelastic properties, such as strain-rate sensitivity [19].

## Declaration of Competing Interest

The authors declare that they have no known competing financial interests or personal relationships that could have appeared to influence the work reported in this paper.
